# Evaluation of PTV margins with daily iterative online adaptive radiotherapy for postoperative treatment of endometrial and cervical cancer: a prospective single-arm phase 2 study

**DOI:** 10.1186/s13014-023-02394-2

**Published:** 2024-01-04

**Authors:** Guangyu Wang, Zhiqun Wang, Yuping Guo, Yu Zhang, Jie Qiu, Ke Hu, Jing Li, JunFang Yan, Fuquan Zhang

**Affiliations:** 1 Department of Radiation Oncology, Peking Union Medical College Hospital, Chinese Academy of Medical Science and Peking Union Medical College, Beijing, 100730 China; 2 Department of Radiation Oncology, State Key Laboratory of Complex Severe and Rare Diseases, Peking Union Medical College Hospital, Chinese Academy of Medical Science and Peking Union Medical College, Beijing, China; 3 Tumor Hospital affiliated to Xinjiang Medical University, Urumqi, China; 4 Varian, a Siemens Healthineers Company, Palo Alto, CA USA

**Keywords:** Cone-beam computed tomography, Online adaptive radiotherapy, Margins, Acute toxicity, Endometrial cancer, Cervical cancer

## Abstract

**Background:**

To determine the optimal planning target volume (PTV) margins for adequate coverage by daily iterative cone-beam computed tomography (iCBCT)-guided online adaptive radiotherapy (oART) in postoperative treatment of endometrial and cervical cancer and the benefit of reducing PTV margins.

**Methods:**

Fifteen postoperative endometrial and cervical cancer patients treated with daily iCBCT-guided oART were enrolled in this prospective phase 2 study. Pre- and posttreatment iCBCT images of 125 fractions from 5 patients were obtained as a training cohort, and clinical target volumes (CTV) were contoured separately. Uniform three-dimensional expansions were applied to the PTVpre to assess the minimum margin required to encompass the CTVpost. The dosimetric advantages of the proposed online adaptive margins were compared with conventional margin plans (7–15 mm) using an oART emulator in another cohort of 125 iCBCT scans. A CTV-to-PTV expansion was verified on a validation cohort of 253 fractions from 10 patients, and further margin reduction and acute toxicity were studied.

**Results:**

The average time from pretreatment iCBCT to posttreatment iCBCT was 22 min. A uniform PTV margin of 5 mm could encompass nodal CTVpost in 100% of the fractions (175/175) and vaginal CTVpost in 98% of the fractions (172/175). The margin of 5 mm was verified in our validation cohort, and the nodal PTV margin could be further reduced to 4 mm if ≥ 95% CTV coverage was predicted to be achieved. The adapted plan with a 5 mm margin significantly improved pelvic organ-at-risk dosimetry compared with the conventional margin plan. Grade 3 toxicities were observed in only one patient with leukopenia, and no patients experienced acute urinary toxicity.

**Conclusion:**

In the postoperative treatment of endometrial and cervical cancer, oART could reduce PTV margins to 5 mm, which significantly decrease the dose to critical organs at risk and potentially lead to a lower incidence of acute toxicity.

**Supplementary Information:**

The online version contains supplementary material available at 10.1186/s13014-023-02394-2.

## Introduction

For endometrial cancer and early-stage cervical cancer treated with surgery, adjuvant pelvic radiotherapy with or without chemotherapy is prescribed, depending on clinicopathological prognostic factors [1–4]. Previous studies have demonstrated that postoperative radiotherapy could decrease locoregional recurrence and improve survival for patients with high-risk factors [5, 6]. Compared with standard four-field radiotherapy, intensity-modulated radiation therapy (IMRT) has been widely used in pelvic radiotherapy for gynecologic malignancies, with greater conformity in treatment volumes, more homogeneous dose distribution and sharper dose reduction in adjacent organs at risk (OAR) [7–9]. However, the expansion of planning target volume (PTV) margins from IMRT to generate a large target volume needs to encompass both inter- and intrafractional variations, particularly large anatomical variations in the pelvic area, such as bladder and rectal filling. Postoperative pelvic radiotherapy with large target volumes may result in acute gastrointestinal and genitourinary toxicity [10, 11].

Recently, commercially available online adaptive radiotherapy (oART) has allowed automatic target volume delineation and per-fraction treatment plan reoptimization, potentially improving dosimetric outcomes [12, 13]. Further reduction of PTV margins may be possible by adapting the treatment to the patient’s daily anatomy. However, a certain intrafractional uncertainty should be considered for PTV expansions in oART. The time required to adapt anatomical variations and replan treatment cannot be ignored, especially the impact of the volumes of the bladder and rectum, which vary randomly during this time. A fast iterative cone-beam computed tomography (iCBCT)-guided daily oART technique showed great advantages in terms of both adaptive time and online workflow. Currently, it is not clear to what extent daily oART reduces PTV margins for postoperative cervical cancer and endometrial cancer, and the clinical benefits of reducing PTV margins have not been reported in previous studies.

We performed a prospective phase 2 study of daily oART for postoperative treatment of endometrial and cervical cancer to determine the optimal PTV margins for adequate coverage and to investigate the dosimetric and clinical benefits of reduced PTV margins.

## Methods and materials

### Patient eligibility

Fifteen patients with postoperative endometrial and cervical cancer treated with daily iCBCT-guided oART were enrolled in this prospective nonrandomized phase 2 trial between September 2022 and February 2023. Eligible patients underwent surgical resection and had indications for adjuvant pelvic radiotherapy. The full eligibility and exclusion criteria are summarized in Supplementary Table A1. This study was reviewed and approved by the Institutional Review Board of Peking Union Medical College Hospital (No. K2416), and the registration code of the trial (ClinicalTrials.gov ID) is NCT05682950.

### Target delineation and treatment

Delineation of clinical target volume (CTV) was performed according to the NRG Oncology/RTOG Consensus Guidelines [14], consisting of separate nodal CTV (CTV-N) and vaginal CTV (CTV-V) contours. The anterior border of CTV-V is the posterior aspect of the bladder wall, and the posterior border is the anterior rectal wall, including approximately the anterior one-third of the mesorectum, and the obturator nodal CTV is carved out of bladder, which does not take interfractional organ motion into account.

All patients received postoperative daily iCBCT-guided oART (Ethos, Varian Medical Systems, Palo Alto, USA), and a prescribed dose of 45 or 50.4 Gy in 25 or 28 fractions was applied to the PTV. Before simulation and each treatment fraction, patients were instructed to empty their bladder and rectum one hour and forty minutes before the appointment, followed by an intake of 450–500 ml water in 10 min according to their height and weight. The patients were fixed with a thermoplastic film and simulated in supine position, with their arms above their head or on their chest.

The CT-guided high-dose intracavitary brachytherapy was administered to the upper 1/3 or 1/2 of the vagina after oART, with a prescribed dose of 10 Gy in 2 fractions delivered to a depth of 0.5 cm below the vaginal mucosa.

### iCBCT acquisition and registration

All enrolled patients underwent three iCBCT scans per oART fraction. The adaptive CTV was contoured based on the pretreatment iCBCT (iCBCT-1), which was acquired after patient enrollment. The second iCBCT (iCBCT-2) scan was obtained to verify the position of the target volume and OARs before treatment. The posttreatment iCBCT (iCBCT-3) scan was acquired immediately after treatment completion. The total time from the acquisition of the pretreatment iCBCT to the completion of the posttreatment iCBCT was recorded.

The iCBCT-3 scans were uploaded to the Ethos oART emulator, which was provided by Varian Medical Systems for remote simulation of an Ethos system with identical software and functions as the clinical version, and CTV-Npost and CTV-Vpost were contoured on each iCBCT. Then, iCBCT-3 scans with CTVpost were rigidly registered to the iCBCT-1 scans with PTVpre with respect to bony anatomy using an Eclipse treatment planning system (Varian Inc., Palo Alto, CA). The CTV delineation, iCBCT registration and target coverage evaluation were performed by the same physician and verified by a second expert radiation oncologist.

### Target coverage evaluation from the training cohort

A total of 125 pre- and posttreatment iCBCT scans, corresponding to each fraction from 5 patients with conventional PTV margins (CTV-N expanded 10 mm in the superior-inferior direction and 7 mm in the anterior-posterior and lateral directions and CTV-V uniformly expanded 15 mm), were analyzed as a training cohort. Uniform three-dimensional planning margins of 5, 7, 10, 12, and 15 mm were added to the CTVpre to generate PTVpre, which were then projected onto each posttreatment iCBCT scan to assess the minimum planning margins required to encompass the CTVpost in the anterior-posterior, lateral, and superior-inferior directions.

### Dosimetric evaluation

After determining the minimum adaptive margin expansion based on the training cohort, a separate cohort of 125 iCBCT scans from 5 postoperative endometrial and cervical cancer patients previously treated on Halcyon Linac (Varian Medical Systems, Palo Alto, USA) were evaluated to determine the dosimetric benefit of reduced online adaptive PTV margins. These iCBCT scans were uploaded to the Ethos oART emulator for daily adaptive replanning, and two reference plans on the same fraction were generated, adopting two margins and using the same planning templates. The adaptive margin plan, using the determined adaptive margin (5 mm), was compared with the conventional margin plan.

### Target coverage evaluation from the validation cohort and follow-up

A CTV-to-PTV uniform margin expansion of 5 mm was verified on a validation cohort of 253 pairs of iCBCT scans in 256 fractions from 10 enrolled patients treated with daily oART, and margins of 0, 1, 2, 3, 4, and 5 mm in each of the above directions were explored. The CTV-N was divided into upper, middle and lower portions for precise evaluation, and the boundaries were the bifurcation of the common iliac artery and the appearance of the piriformis muscle.

**Fig. 1:**
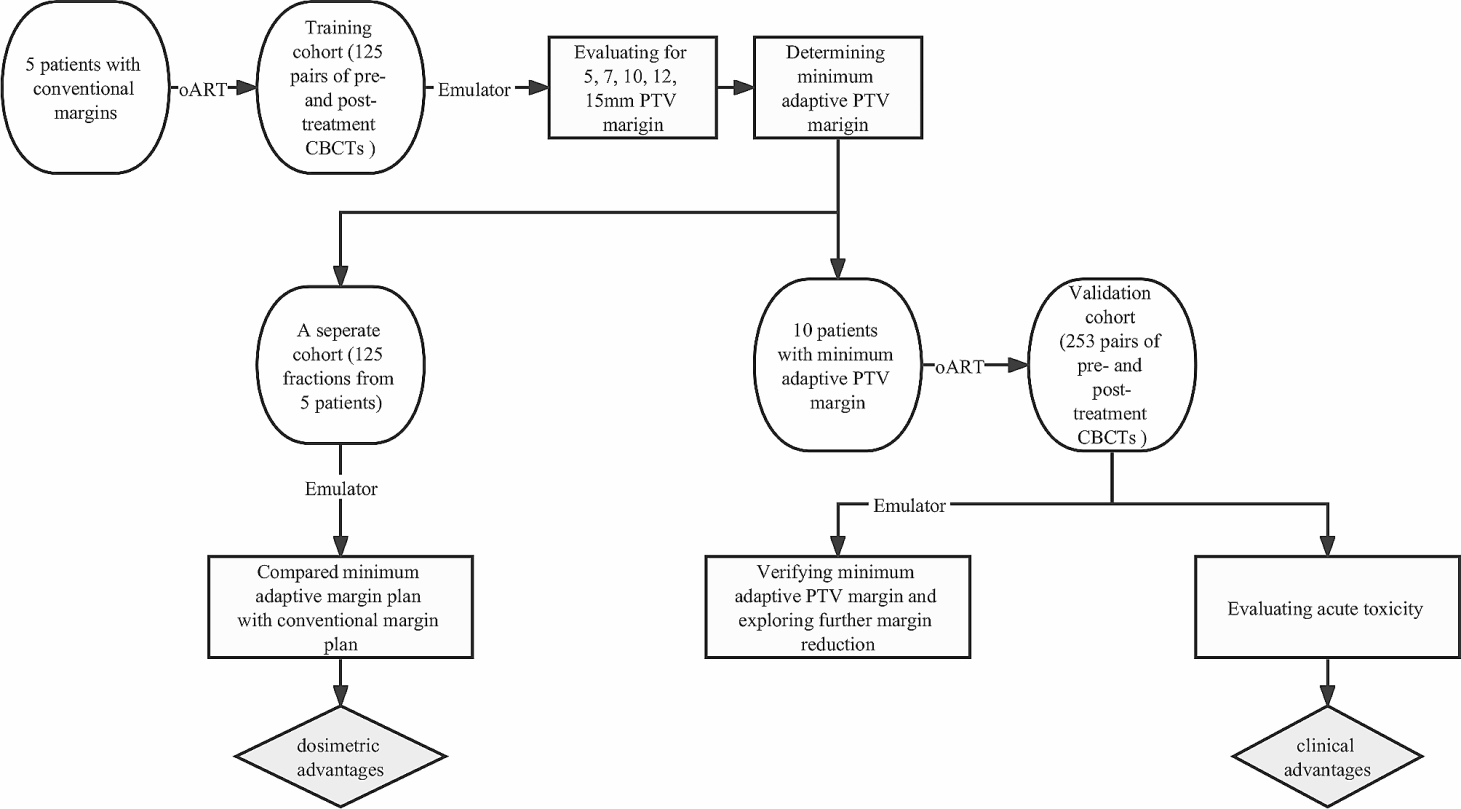
Workflow of this study to determine the optical PTV margins and explore the clinical and dosimetric advantages of reduced margins. The minimum adaptive margin expansion for the training cohort was determined by registering pre- and posttreatment iCBCT images with respect to bony anatomy. A separate cohort with a total of 125 fractions of iCBCT scans was uploaded to the oART simulator for dosimetric advantage evaluation with the minimum adaptive margins. The minimum adaptive expansion was verified in a validation cohort, and acute toxicity was studied

Acute toxicities, measured from the initiation of oART to 90 days after completion, of the patients from the validation cohort with reduced 5 mm PTV margins were studied. Since the beginning of the treatment, patients were interviewed weekly during the period of oART and followed up by evaluations at one-month intervals for three months after completing the treatment. Toxicities were graded according to the National Cancer Institute Common Terminology Criteria for Adverse Events, version 5.0 (CTCAE 5.0). The workflow of this study is presented in Fig. 1.

### Statistical analysis

The statistical analysis was performed using SPSS (version 27.0; IBM Corp). The minimum PTVpre margins to encompass CTVpost were assessed using the chi-square test and compared with CTV 100% coverage. Data with a normal distribution were analyzed by *t* test, and data with a nonnormal distribution or heterogeneous variance were analyzed by nonparametric test. *p* values < 0.05 denoted a significant difference.

## Results

### Clinical characteristics of patients

Five postoperative endometrial cancer patients and ten cervical cancer patients with 381 fractions were enrolled in this prospective study and treated with daily oART. Three fractions for PTV margin analysis were excluded in the validation cohort due to posttreatment iCBCT loss. Supplementary Table A2 summarizes the clinicopathological characteristics of the patients.

### Target coverage in the training cohort

The average time from iCBCT-1 to iCBCT-3 was 22 min 54 s (range: 17 min 24 s − 38 min 43 s). After the posttreatment iCBCT scan was rigidly registered pretreatment iCBCT scan with respect to bony anatomy, a uniform three-dimensional CTV-Npre to PTV-Npre margin of 5, 7, 10, 12, and 15 mm could cover CTV-Npost in all fractions (125/125, *p* > 0.05). A uniform margin of 5 mm could encompass 98% of the CTV-Vpost (123/125, *p* > 0.05), and a 7 mm anterior expansion was needed to cover the remaining two fractions. This 5 mm expansion was then used in the dosimetric evaluation and validation cohort.

### Dosimetric outcomes

Dosimetric outcomes were assessed in a separate cohort of 125 daily fractions of 5 patients. Table 1 shows the complete list of dosimetry comparisons among the adapted plan with 5 mm margin oART (A-ART) and the adapted plan with conventional margin oART (A-CRT), and the target volume for both plans achieved clinically acceptable quality. Bladder dosimetry significantly improved with reduced margins (*p* < 0.05), including V_40Gy_ (A-ART 20.78% vs. A-CRT 35.53%), V_30Gy_ (A-ART 33.94% vs. A-CRT 52.1%), V_20Gy_ (A-ART 54.06% vs. A-CRT 71.81%), V_10Gy_ (A-ART 90.18% vs. A-CRT 91.95%), and D_mean_ (A-ART 0.99 Gy vs. A-CRT 1.22 Gy). The reduced margin also significantly decreased the dose to critical OARs, such as the rectum, bone marrow, left femur head, right femur head and bowel.

**Table 1:** List of dosimetric outcomes for all emulated125 daily fractions of 5 patients

Target and OAR	Goal	Adapted plan, 5 mm margins	Adapted plan, conventional margin	*p value*
CTV-N	V_100%_ (%)	99.59 ± 0.14	99.7 ± 0.19#	*p* < 0.05
CTV-V	V_100%_ (%)	99.54 ± 0.30	99.85 ± 0.15#	*p* < 0.05
PCTV-N	V_100%_ (%)	95.90 ± 0.20	96.04 ± 0.55#	*p* < 0.05
PCTV-V	V_100%_ (%)	95.47 ± 0.81	95.51 ± 1.46	*p* = 0.801
Bladder	V_40Gy_ (%)	20.78 ± 4.29	35.53 ± 6.36#	*p* < 0.05
	V_30Gy_ (%)	33.94 ± 5.64	52.10 ± 7.10#	*p* < 0.05
	V_20Gy_ (%)	54.06 ± 6.94	71.81 ± 6.84#	*p* < 0.05
	V_10Gy_ (%)	90.18 ± 5.81	91.95 ± 5.27#	*p* < 0.05
	D_mean_ (Gy)	0.99 ± 0.08	1.22 ± 0.09#	*p* < 0.05
Rectum	V_40Gy_ (%)	38.27 ± 7.66	80.35 ± 9.11#	*p* < 0.05
	V_30Gy_ (%)	59.42 ± 8.52	89.34 ± 6.59#	*p* < 0.05
	V_20Gy_ (%)	75.49 ± 7.60	95.20 ± 4.29#	*p* < 0.05
	V_10Gy_ (%)	94.06 ± 4.14	99.29 ± 1.45#	*p* < 0.05
	D_mean_ (Gy)	1.26 ± 0.10	1.67 ± 0.08#	*p* < 0.05
Bone Marrow	V_40Gy_ (%)	14.76 ± 1.53	21.36 ± 2.06#	*p* < 0.05
	V_10Gy_ (%)	76.88 ± 2.24	81.08 ± 2.74#	*p* < 0.05
	D_mean_ (Gy)	0.88 ± 0.03	0.99 ± 0.04#	*p* < 0.05
	D_90%_ (Gy)	0.20 ± 0.03	0.26 ± 0.04#	*p* < 0.05
Femur head left	V_30Gy_ (%)	0.52 ± 0.74	1.94 ± 1.71#	*p* < 0.05
	D_mean_ (Gy)	0.45 ± 0.03	0.50 ± 0.04#	*p* < 0.05
	D_5%_ (Gy)	0.82 ± 0.08	0.96 ± 0.14#	*p* < 0.05
Femur head right	V_30Gy_ (%)	0.51 ± 0.67	1.77 ± 1.77#	*p* < 0.05
	D_mean_ (Gy)	0.45 ± 0.02	0.50 ± 0.04#	*p* < 0.05
	D_5%_ (Gy)	0.84 ± 0.06	0.94 ± 0.13#	*p* < 0.05
Bowel	V_40Gy_ (%)	13.26 ± 3.3	18.32 ± 4.63#	*p* < 0.05
	V_30Gy_ (%)	27.45 ± 4.82	33.39 ± 5.82#	*p* < 0.05
	V_20Gy_ (%)	50.98 ± 4.1	54.78 ± 5.38#	*p* < 0.05
	V_10Gy_ (%)	75.14 ± 4.90	77.84 ± 6.30#	*p* < 0.05
	D_2cm3_ (Gy)	1.86 ± 0.01	1.89 ± 0.01#	*p* < 0.05
	V_40Gy_ (cm^3^)	87.56 ± 24.32	121.0 ± 33.29#	*p* < 0.05
	V_47Gy_ (cm^3^)	0.95 ± 0.58	2.5 ± 2.13#	*p* < 0.05

Abbreviations: OAR, organs at risk

Note: Results are presented as the average value together with one standard deviation. *p* value represents the outcome of *t* test

# Adapted plan with 5 mm margins compared with adapted plan with conventional margin, *p* < 0.05

### Target coverage in the validation cohort

**Fig. 2:**
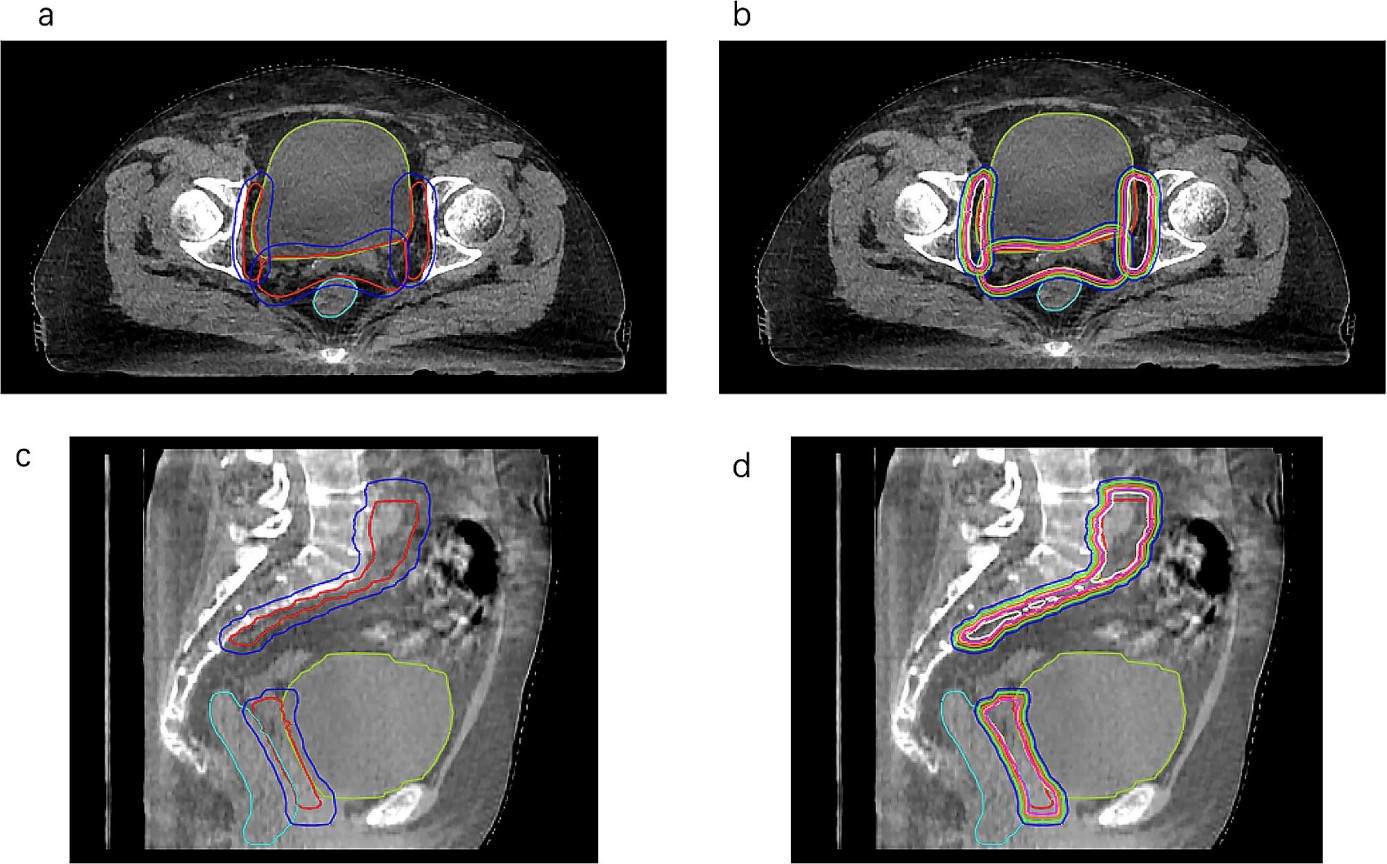
Example images of axial (**a** and **b**) and sagittal (**c** and **d**) slices of a patient from the validation cohort. The CTVpost (red) and PTVpre (blue) contours were superimposed onto the images after pre- and posttreatment iCBCT matching with respect to bony anatomy. After 100% coverage with a 5 mm margin (a and c), margins of 0 (CTVpre, yellow), 1 (white), 2 (magenta), 3 (orange), 4 (green), and 5 (PTVpre, blue) mm in the three-dimensional direction were explored (b and d)

The average time from iCBCT-1 to iCBCT-3 was 22 min 47 s (range: 18 min 15 s -26 min 46 s). Figure 2 shows the CTVpost and PTVpre contours superimposed onto the images after pre- and posttreatment iCBCT matching to exemplify the required minimum margin. Using a uniform 5-mm expansion, 100% (253/253, *p* > 0.05) of CTV-Npost was covered, and 99% (250/253, *p* > 0.05) of CTVpost was covered. The three fractions of two patients not fully covered needed the following minimum margins to cover the difference: 7 mm in the anterior direction; 6 mm in the anterior, posterior, left directions; 8 mm in the posterior direction, and 7 mm in the left direction. After further isocentric reduction of the PTV margins to 4 mm, the percentage of CTV-N coverage was greater than 91.3% (231/253) in six directions. This was not statistically significant (*p* > 0.05) if ≥ 95% CTV coverage was predicted to be achieved. The margins to encompass the CTVpost in the anterior-posterior, lateral, and superior-inferior directions are shown in Table 2.

**Table 2:** The margins to encompass the CTVpost in anterior-posterior, lateral, and superior-inferior directions

CTV	Margin	Directions					
		Anterior (N,%)	Posterior (N,%)	Left (N,%)	Right(N,%)	Superior (N,%)	Inferior (N,%)
CTV-N (upper)	≤ 1 mm	1 (0.4) *	0*	0*	1(0.4) *	47 (18.6) *	-
	≤ 2 mm	9 (3.6) *	5 (2) *	9 (3.6) *	22 (8.7) *	114 (45.1) *	-
	≤ 3 mm	154 (60.9) *	199 (78.7) *	189 (74.7) *	206 (81.4) *	226 (89.3) *	-
	≤ 4 mm	237 (93.7)	241 (95.3)	243 (96.0)	246 (97.2)	246 (97.2)	-
	≤ 5 mm	253 (100)	253 (100)	253 (100)	253 (100)	253 (100)	-
CTV-N (middle)	≤ 1 mm	0*	0*	0*	1 (0.4) *	-	-
	≤ 2 mm	4 (1.6) *	7 (2.8) *	6 (23.7) *	9 (3.6) *	-	-
	≤ 3 mm	145 (57.3) *	218 (86.2) *	205 (81) *	217 (85.8) *	-	-
	≤ 4 mm	232 (91.7)	249 (98.4)	242 (95.7)	247 (97.6)	-	-
	≤ 5 mm	253 (100)	253 (100)	253 (100)	253 (100)	-	-
CTV-N (lower)	≤ 1 mm	0*	0*	0*	0*	-	37 (14.6) *
	≤ 2 mm	17 (6.7) *	5 (2) *	2 (0.8) *	1 (0.4) *	-	135 (53.4) *
	≤ 3 mm	172 (70) *	179 (70.8) *	158 (62.5) *	167 (66.0) *	-	238 (94.1)
	≤ 4 mm	234 (92.5)	239 (94.5)	236 (93.3)	241 (95.3)	-	251 (99.2)
	≤ 5 mm	253 (100)	253 (100)	253 (100)	253 (100)	-	253 (100)
CTV-V	≤ 1 mm	5 (2) *	3 (1.2) *	1 (0.4) *	3 (1.2) *	66 (26.1) *	67 (26.5) *
	≤ 2 mm	31 (12.3) *	17 (6.7) *	26 (10.3) *	27 (10.7) *	129 (51) *	146 (57.7) *
	≤ 3 mm	121 (47.8) *	91 (40) *	159 (62.8) *	159 (62.8) *	224 (88.5) *	223 (88.1) *
	≤ 4 mm	193 (76.3) *	184 (72.7) *	216 (85.4) *	220 (87) *	238 (94.1)	236 (93.3)
	≤ 5 mm	251 (99.2)	251 (99.2)	252 (99.6)	252 (99.6)	253 (100)	253 (100)

Abbreviations: CTV, clinical target volume

Note: * refers to the statistically difference (*p* < 0.05) of this margin compared with CTV 95% coverage at one-sided test

### Acute toxicities

The follow-up data from ten patients with reduced 5 mm margins were complete. No patient experienced distant metastasis or pelvic recurrence at the last follow-up. Table 3 shows the acute toxicities. No urinary or skin toxicities were observed in this study. Leukopenia (6/10 patients) was the most common hematologic toxicity, while diarrhea (4/10 patients) and nausea (3/10 patients) were the most common gastrointestinal disorders. One patient had mild vaginal discharge, and one had slightly increased alanine aminotransferase levels. Toxicities were relieved in all but one patient with grade 1 diarrhea at one month after the end of oART. No toxicity was reported at the three-month and six-month follow-up.

**Table 3:** Acute treatment-related toxicities

Acute toxicities	Grade 1	Grade 2	Grade 3	Grade 4
*During treatment*				
Hematologic	1 (10%)	4 (10%)	1 (10%)	0
GI	2 (20%)	4 (40%)	0	0
Urinary	0	0	0	0
Dermatitis	0	0	0	0
Genital	1 (10%)	0	0	0
Laboratory test for hepatobiliary disorders	1 (10%)	0	0	0
Malaise	2 (20%)	1 (10%)	0	0
*End of treatment to one months*				
GI	1 (10%)	0	0	0
*End of treatment to three months*				
Disorders	0	0	0	0

Abbreviations: GI, gastrointestinal

Note: Grading is reported as the maximum symptoms at the trial time points

## Discussion

To our knowledge, this is the first prospective clinical study to evaluate PTV margins for postoperative treatment of endometrial and cervical cancer with daily oART. The PTV margins and OAR volumes were correlated with the severity of irradiation toxicity [15, 16]. Traditional PTV margins for postoperative endometrial and cervical cancer have been large, and the recommendation is to have a uniform expansion of 5 to 10 mm for CTV-N and an expansion of 6 to 8 mm for CTV-V if using an ITV with daily CBCT guidance [14]. In the NRG/Radiation Therapy Oncology Group (RTOG) 1203 trial, a uniform PTV expansion of 7 mm was used on the basis of the ITV determined by emptying and filling the bladder [17]. Image-guided irradiation therapy (IGRT) allows real-time monitoring of the target and OAR but cannot conduct interfractional interventions, such as modifying the target volume influenced by bladder and rectum filling. In contrast, daily oART may potentially allow for reduced CTV-to-PTV margins by adapting to the deforming operative bed. However, the extent and value of margin reduction must be determined and validated in clinical trials.

In a previous similar experiment, the predicted margins were estimated by equations comparing the overlap of CTVpost and PTVpre to evaluate adequate coverage [18, 19]. Using equations to calculate the predicted margin may yield better coverage, but there may be some areas in each fraction that are not covered, and whether this missed area has a clinical impact is unclear. In this prospective study, we adopted more stringent judgment criteria to assess target coverage, according to Jhingran et al. [20], evaluating the PTV margins with 100% morphological overlap of CTV coverage. In addition, unlike most previous weekly adaptation studies [17, 18], our department appointed designated physicians, physicists and therapists to participate in this phase 2 study concerning daily adaptation. Given that, we anticipate that the current study would be more accurate. As this was the first clinically implemented online adaptive workflow with postoperative cervical and endometrial cancer with no defined PTV margins, we started with a conventional margin (7–15 mm) in the training cohort. Then, posttreatment iCBCT scans were uploaded to the oART emulator to determine the expansion margins in the validation cohort.

In previous studies, CTV-V has received more research attention and is considered to be more prone to variation than CTV-N due to the daily changes in the operative bed caused by the adjacent bladder and rectum pushing boundaries. Jhingran et al. [21] evaluated vaginal vault variations during a 5-week course of postoperative radiotherapy of endometrial and cervical cancer through measured movement of vaginal markers, and they found that the maximum variations were largest in the anterior-posterior and superior-inferior directions, with a median of 1.46 cm and 1.2 cm, respectively, and the median maximum movement was 0.59 in the right-left direction. Jürgenliemk-Schulz et al. [22] reported that the CTV-V position changed after hysterectomy for cervical or endometrial cancer during the course of radiotherapy. Homogenous CTV-to-PTV margins that allowed complete coverage of 90% and 95% of CTV-V were 0.9–1.1 cm and 2.3–1.5 cm, respectively, and CTV-V was not completely covered in 53.3% and 20.0% of fractions with homogenous CTV–PTV margins of 1.0 and 1.5 cm, respectively. These studies performed offline adaptive analysis using weekly MR or CT scans during treatment, which could not represent the intrafractional changes and were therefore larger than what we reported in our study. In contrast, our results confirmed that daily oART could correct these variations and significantly reduce PTV margins; specifically, the borders of CTV-V were the posterior bladder wall and the anterior rectal wall, which does not take interfractional organ motion into account. The missed CTV-V area occurred mostly in the anterior-posterior direction for fractions with 5-mm margins, which is consistent with the maximum anterior–posterior shift reported above.

Yen et al. [19] first performed CTV delineation on pre- and posttreatment CBCT scans to analyze the PTV margin for the cervical lesion region by using CBCT-guided oART, and they recommended that a 5 mm CTV expansion was sufficient for cervical lesions without complex circumstances. However, they did not explore margins for CTV-N. Our results indicated that the PCTV-N margins could be further reduced to 4 mm (*p* > 0.05) if ≥ 95% CTV coverage was predicted to be achieved. However, the PTV-N margin could not be further reduced, which may be inconsistent with our initial assumption that CTV-N is close to the pelvis and that the ITV could be corrected after iCBCT registration with respect to bony anatomy, resulting in further reductions in CTV-N expansion margins. These discrepancies could be from a residual rotational error, which could not be ignored in daily CBCT-guided radiotherapy in the study by Laursen and colleagues [23], and Ethos Linac from our study is equipped with a three-dimensional treatment couch that could correct limited rotational error. Furthermore, the observer bias for CTV contour inevitably increased as the PTV margin decreased. Our results also indicated that the PTV margins in the anterior-posterior direction of the middle CTV-N and lateral direction of the lower CTV-N were smaller than those in other directions in the same regions. Given that the presacral nodal CTV was not contoured when the piriformis muscle appeared, the vertical displacement of the image easily leads to missed CTV in the anterior-posterior direction with small PTV margins after pretreatment iCBCT was rigidly matched to posttreatment iCBCT with respect to bony anatomy. Another potential explanation is that the degree of relaxation of the piriformis muscle changed before and after treatment, which affected the delineation of CTV. In the lower CTV-N portion, the obturator nodal CTV is affected by intrafractional bladder filling, which may account for the larger lateral expansion margins of the lower CTV-N.

The implementation of reduced margins in our study resulted in a significantly lower OAR dose. This is consistent with previous studies that have demonstrated the dosimetric advantages of reduced margins compared with larger conventional margins [13, 24, 25]. These dosimetric advantages ultimately need to be reflected in clinical advantages. When compared with previous studies [17, 26, 27], the acute complication rates observed in our enrolled patients were more satisfactory, with no grade 3–4 adverse gastrointestinal complications and no urinary complications, correlating well with the improvement in bladder dosimetry. However, the improvement of grade 1 and 2 gastrointestinal toxicity and hematological toxicity was not obvious, which was not consistent with the significantly reduced dose to the corresponding OAR. This may be related to the resulting high toxicity rates from the majority of enrolled patients receiving concurrent chemotherapy or prior chemotherapy. Furthermore, although the posterior border was the anterior rectal wall and interfractional organ motion was not considered when the observer delineated CTV-V, the residual uterosacral ligaments and approximately the anterior one-third of the mesorectum still needed to be contoured. After expansion with a 5 mm PTV margin, most of the rectum was still irradiated.

Given that the coverage of the para-aortic lymph node chain required the use of extended iCBCT and was time-consuming, patients who had indications for treatment with extended-field pelvic radiotherapy were not enrolled in this study, so the PTV-N margins for the para-aortic nodal CTV were not evaluated. In addition, considering the implementation efficiency and time span of this clinical trial, a separate cohort of 125 iCBCT scans from Halcyon Linac was uploaded to the oART simulator for dosimetric advantage evaluation after determining the minimum margin required to encompass the CTVpost instead of using the iCBCT scans from the validation cohort. Furthermore, the PTV expansions in oART data presented in this study were based on 378 fractions from 15 patients. This is sufficient for evaluating PTV margins but far from sufficient to assess irradiation toxicities, which need to be studied in larger populations.

## Conclusion

In summary, to our knowledge, this is the first prospective phase 2 study to evaluate PTV margins for postoperative oART of endometrial and cervical cancer. The oART margins could be reduced to 5 mm symmetrically while maintaining excellent dosimetric coverage, and oART with these reduced margins could significantly decrease the dose to OARs and lead to a lower incidence of acute toxicity. Furthermore, PTV-N expansion could be further narrowed with strict bladder and bowel preparations. Currently, the PTV margins reduction could only be safely accomplished with daily oART, and requires favorable logistics to accommodate prolonged treatment time on couch. The irradiation toxicity of oART will need to be validated prospectively in a larger cohort.

### Electronic supplementary material

Below is the link to the electronic supplementary material.

**Supplementary Material 1: Table A1** Study inclusion and exclusion criteria. **Table A2** Clinicopathological characteristics

## Data Availability

All data generated or analysed during this study are included in this published article.

## Abbreviations

CTCAE: National Cancer Institute Common Terminology Criteria for Adverse Events
CTV-N: Nodal clinical target volume
CTV-V: Vaginal clinical target volume
iCBCT: Iterative cone-beam computed tomography
IMRT: Intensity-modulated radiation therapy
ITV: Internal target volume
OAR: Organ at risk
oART: Online adaptive radiotherapy
PTV: Planning target volume
