# Supplementary material for: Evaluation of PTV margins with daily iterative online adaptive radiotherapy for postoperative treatment of endometrial and cervical cancer: a prospective single-arm phase 2 study
Source: Radiat Oncol. 2024 Jan 4;19:2. doi: 10.1186/s13014-023-02394-2 (PMC10768299; doi:10.1186/s13014-023-02394-2)
Supplement: Supplementary file 1 — Supplementary Material 1: Table A1 Study inclusion and exclusion criteria. Table A2 Clinicopathological characteristics [file 13014_2023_2394_MOESM1_ESM.docx]

**Table A1** Study inclusion and exclusion criteria

| Inclusion criteria |  | |
| --- | --- | --- |
| aged ≥ 18 years and ≤ 70 years | | |
| Karnofsky score ≥ 70 | | |
| Pathological findings indicate risk factors and adjuvant radiotherapy are prescribe | | |
| No residual tumor at the resection margin with pathologically confirmed | | |
| No evidence of distant metastasis (FIGO stage IVB) | | |
| No evidence of para-aortic metastatic lymph nodes | | |
| No contraindications to CT scanning | | |
| Provision of written informed consent before treatment | | |
| Tolerate online adaptive time | | |
| Exclusion criteria | |  |
| Previous irradiation to the pelvic region | | |
| With other primary malignancies | | |
| Severe disease which may bring unacceptable risk or affect the compliance of the trial | | |
| Active inflammatory bowel disease | | |

Abbreviations: FIGO: International Federation of Gynecology and Obstetrics.

**Table A2** Clinicopathological characteristics

| Characteristics | Number (n) | Percent (%) |
| --- | --- | --- |
| *Median age (year)* | 49 |  |
| *Age range (year)* | 31-69 |  |
| *Primary tumor* | 15 | 100 |
| Endometrial | 5 | 33 |
| Cervical | 10 | 67 |
| *FIGO Staging of endometrial* | 5 | 100 |
| IA | 1 | 20 |
| IB | 2 | 40 |
| IIIB | 1 | 20 |
| IIIC1 | 1 | 20 |
| *FIGO Staging of cervical* | 10 | 100 |
| IB1 | 2 | 20 |
| IB2 | 5 | 50 |
| IB3 | 1 | 10 |
| IIIC1 | 2 | 20 |
| *Pat**hological classification of cervical cancer* | 10 | 100 |
| Adenocarcinoma | 2 | 20 |
| Squamous | 6 | 60 |
| Adenosquamous | 2 | 20 |
| *Prescribed dose and fractions* | 15 | 100 |
| 45Gy/25f | 13 | 87 |
| 50.4Gy/28f | 2 | 13 |
| *Concurrent chemotherapy* | 15 | 100 |
| Yes | 6 | 40 |
| No | 9 | 60 |
| *Previous chemotherapy* | 15 | 100 |
| Yes | 2 | 13 |
| No | 13 | 87 |

Abbreviations: FIGO: International Federation of Gynecology and Obstetrics; CTV, clinical target volume.
